# Surface wear in a custom manufactured temporomandibular joint prosthesis

**DOI:** 10.1002/jbm.b.35010

**Published:** 2022-01-28

**Authors:** Nikolas De Meurechy, Merve Kübra Aktan, Bart Boeckmans, Stijn Huys, Denis R. Verwilghen, Annabel Braem, Maurice Y. Mommaerts

**Affiliations:** ^1^ European Face Centre Universitair Ziekenhuis Brussel Brussels Belgium; ^2^ Doctoral School of Life Sciences and Medicine Vrije Universiteit Brussel Brussels Belgium; ^3^ Department of Materials Engineering KU Leuven Heverlee Belgium; ^4^ Department of Mechanical Engineering KU Leuven Heverlee Belgium; ^5^ Flanders Make Heverlee Belgium; ^6^ Sydney School of Veterinary Sciences, Faculty of Science University of Sydney Sydney Australia

**Keywords:** animal, arthroplasty, replacement, models, temporomandibular joint, titanium

## Abstract

The wear of a novel temporomandibular joint (TMJ) prosthesis was evaluated in an animal model. The prosthesis consisted of an additively manufactured titanium alloy (Ti_6_Al_4_V) mandibular condyle and glenoid fossa created through selective laser melting, with a machined vitamin E‐enriched ultra‐high molecular weight polyethylene (UHMWPE) surface attached to the fossa. Thirteen TMJ prosthesis were implanted in sheep, six of which had condylar heads coated with HadSat® diamond‐like carbon (H‐DLC). Euthanasia took place after 288 days, equaling 22 years of human mastication. Linear and volumetric wear analysis of the fossa was performed by optical scanning. The condylar head surfaces were assessed by scanning electron and confocal laser microscopy. The average linear UHMWPE wear, when combined with the coated condyle, was 0.67 ± 0.28 mm (range: 0.34–1.15 mm), not significantly differing (*p* = .3765, *t*‐test) from the non‐coated combination average (0.88 ± 0.41 mm; range: 0.28–1.48 mm). The respective mean volumetric wear volumes were 25.29 ± 11.43 mm^3^ and 45.85 ± 22.01 mm^3^, not significantly differing (*p* = .1448, *t*‐test). Analysis of the coated condylar surface produced a mean Ra of 0.12 ± 0.04 μm and Sa of 0.69 ± 0.07 μm. The non‐coated condylar surface measured a mean Ra of 0.28 ± 0.17 μm and Sa of 2.40 ± 2.08 μm. Both Sa (*p* = .0083, Mann–Whitney *U* test) and Ra (*p* = .0182, Mann–Whitney *U* test), differed significantly. The prosthesis exhibits acceptable wear resistance and addition of the H‐DLC‐coating significantly improved long‐term condylar surface smoothness.

## 1 INTRODUCTION

Since the first implantation of alloplastic material as a means to treat temporomandibular joint (TMJ) disease, many different prosthetic concepts have been developed, including a total joint replacement (TJR) with both condylar and fossa component. Although the indications for TMJ TJR remain limited, an increase in case numbers has been observed in recent years―including younger patients^1^, ^2^, ^3^―raising concerns about the lifespan of the used prostheses. A recent meta‐analysis on total knee prostheses reported 95% and 92% survival of the implant at 15 and 20 years, respectively. They concluded that, when the patient is first treated at a young age, at least one replacement surgery in a patient's lifetime might be necessary.^4^ This is of significant importance, as the expected lifespan of a TJR decrease is inversely correlated to the number of revision surgeries.^5^ The rate at which wear appears can be influenced by both material‐related factors such as material choice, surface roughness, and the geometry of the articulating surfaces, as well as patient related factors such as the amount of force that is generated and the amount of activity and movement.^6^ Wear debris also can lead to foreign‐body giant cell reactions, bone resorption, and aseptic implant loosening, contributing to long‐term implant failure.^2^, ^7^, ^8^, ^9^

Despite several TMJ systems being available on the market, there is a clear lack of both proper in vivo and in vitro wear analysis.^10^, ^11^ This lack of testing is a significant shortcoming, as mechanical properties and wear resistance play a pivotal role in determining the long term outcomes of TJR and, therefore, the need for revision or replacement surgery.^2^, ^7^ As far as the authors are aware of, Van Loon et al.^12^, ^13^ are the only group to publish their in vitro TMJ TJR wear results, prior to commercial release of their prosthesis. They designed a wear testing machine, which simulated the articulation of the mandibular head against the ultra‐high molecular weight polyethylene (UHMWPE) disc, while the implant was submerged in bovine fetal calf serum, diluted with distilled water. The UHMWPE disc was weighed both before and after a 7 million cycle‐run, which corresponds to 10 years in vivo functioning, resulting in a wear rate of 0.65 mm^3^/year or linear wear of less than 0.01 mm/year.^13^ While they afterwards also conducted an in vivo sheep experiment, reporting on histological findings of the peri‐articular tissues, no evaluation of the amount of wear was reported on.^12^ Several more studies evaluated either the histological reaction of the peri‐articular tissues or wear pattern in explanted TMJ TJR after, yet as far as we are aware of, this is the first study to report on TMJR wear results through an in vivo experiment.^9^, ^14^

While wear can be evaluated via either in vitro or in vivo testing, in vivo testing is preferred for TMJ replacements for at least three reasons. Firstly, there is evidence from hip joint prostheses that in vivo wear rates are much higher than those evaluated by in vitro testing, risking underestimation of the total wear rate.^15^ Secondly, the TMJ makes rotational and anteroposterior as well as mediolateral translative movements. Mimicking in vivo scenarios in an in vitro testing environment that captures the specific degrees of freedom in movement that occur during mastication would be extremely difficult. Thirdly, the amount of force to which the TMJ is subjected remains uncertain,^14^, ^16^ which limits the ability to create a reliable in vitro experimental environment. When evaluating potential in vivo animal models, the primate TMJ is most similar to a human's, yet their daily mastication rate is rather low. In addition, several major ethical issues and cost of care prohibit the use of primates for this type of research. It is for said reason that several different animal models, such as the pig, goat, and sheep model, have been investigated and proven reliable and relatable in vivo experimental models for TMJ investigations. While having both their advantages (the anatomically and biomechanical resemblance to the human TMJ) and limitations (the more outspoken laterotrusive movements) both the goat and sheep model are considered the “gold standard” in large animals.^17^, ^18^ Further, sheep spend on average 4 h per day eating at a rate of 128 mastication cycles per minute and an average of 8–9 h per day ruminating at a rate of 100 cycles per minute.^19^ Due to this high daily mastication rate, exceeding that of goats, the total duration of an in vivo evaluation of implant wear can be conducted over a shorter period than in humans or other species.

After the developing of a novel patient‐specific additively manufactured (AM, also referred to as 3D‐printed) titanium (Ti) alloy TMJ replacement system by CADskills BV, which aims to restore laterotrusive movement through reinsertion and integration of the lateral pterygoid muscle (LPM), a sheep model animal experiment was designed to further investigation.^2^, ^20^, ^21^, ^22^, ^23^ Whereas the proper implant integration and LPM insertion was previously evaluated,^24^ this paper aims to evaluate the in vivo wear rate in the condylar and fossa components. Furthermore, the difference in wear between the polished condylar head, coated with a HadSat® diamond‐like carbon (H‐DLC) layer, was investigated and compared to that of the non‐coated condylar head. In addition, the amount of wear of the fossa composed of a machined Vitamin E‐enriched and ɣ‐irradiated UHMWPE component articulating with either type of condylar surface was evaluated. The total evaluation period in the present study was 288 days, which is equivalent to 22 years of human masticatory function.^25^

## 2 MATERIALS AND METHODS

### 2.1 In vivo test subjects

This study was approved by the ethical committee at Medanex Clinic (license number LA 1210576 ‐ code of approval EC MxCl 2018‐090).

Fourteen ewes (Swifter crossbreed) aged 2–5 years, with an average weight of 73.4 kg (range: 52–86 kg) and without any missing teeth were enrolled in the study. They were allowed to roam freely in the meadow until the operation.

First, a pilot surgery was performed on two sheep, consisting of a sham surgery with surgical TMJ approach, including opening of the joint capsule, but without condylectomy or prosthesis implantation in one sheep. The second sheep received a TMJ TJR to establish standard procedures before the following twelve sheep were operated.

During the first post‐operative week, the sheep were kept in solitary confinement, after which they were put together in a larger indoor pen.

### 2.2 Implant manufacturing

Six weeks before surgery, each sheep was subjected to a computed tomography (CT) scan of the head. This data was provided to the engineers of CADskills BV in DICOM format. Using the derived standard template library (STL) files, a virtual condylectomy was performed on the left side, from which a total joint prosthesis was designed using Geomagic Freeform Plus (3D Systems). The overall design of the prosthesis was slightly enhanced for animal use after examining a 3D‐printed plastic model of the first sheep's skull (Makerbot, MakerBot Industries and Formlabs II, Formlabs). However, the actual prosthetic design, including the number of screws and screw diameters, was devised to be similar to those used in humans. The specific course of the inferior alveolar nerve in the sheep was taken into account for screw length and position in the mandibular stump.

**FIGURE 1:**
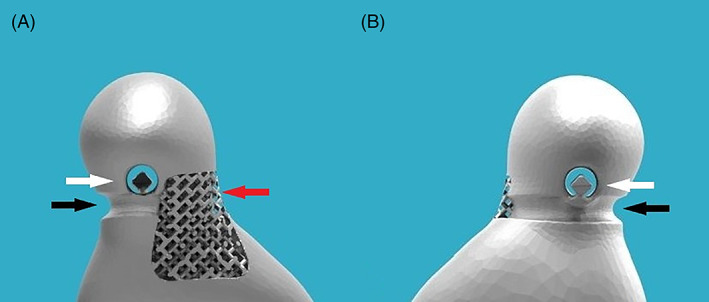
Condylar head with suture threading tunnel and hook for fixation of the lateral pterygoid muscle enthesis. (A) Mesial side. Black arrow: subcondylar groove to guide enthesis' sutures. White arrow: 2.4 mm subcondylar tunnel and hook‐like extension for fixation of the enthesis. Red arrow: lattice structure for enthesis' bony ingrowth. (B) Lateral side. Black arrow: subcondylar groove to guide enthesis' sutures. White arrow: 2.4 mm subcondylar tunnel and hook‐like extension for fixation of the enthesis

The ramal component was produced in a medical‐grade Ti alloy (Ti_6_Al_4_V ELI grade 23) by AM, more specifically selective laser melting (SLM 125 HL, SLM Solutions Group AG). A scaffold structure (500 μm interconnected pores with a diamond unit cell structure) was provided both at the boney interface with the mandible as well as at the condylar neck to provide optimal conditions for boney union and enthesis reconstruction of LPM respectively. A narrow tunnel with a diameter of 2.4–2.5 mm, to accommodate a size 0 suture, was designed in the neck of the condyle (Figure 1). After printing, all condylar heads were first milled to achieve a 0.02 mm accuracy to the “design‐STL”, after which they are polished using a chalk‐based polishing paste. Six of the 13‐condylar heads were further treated with a H‐DLC coating using the non‐disclosed HadSat® protocol, whereas the other seven condylar heads were left untreated after polishing. The identity of the supplier, as well as the means for applying the H‐DLC‐coating onto the condylar head surface are proprietary information. The surface roughness of one, non‐implanted, coated condyle was determined using a confocal laser microscope (Ra = 0.09 μm, Rt = 0.53 μm) to serve as a comparison for the explanted condyles.

Ti alloy screws (Ti_6_Al_4_V grade 5, 2.3 mm diameter; Surgi‐Tec NV) were used for fixation of the ramal component.

**FIGURE 2:**
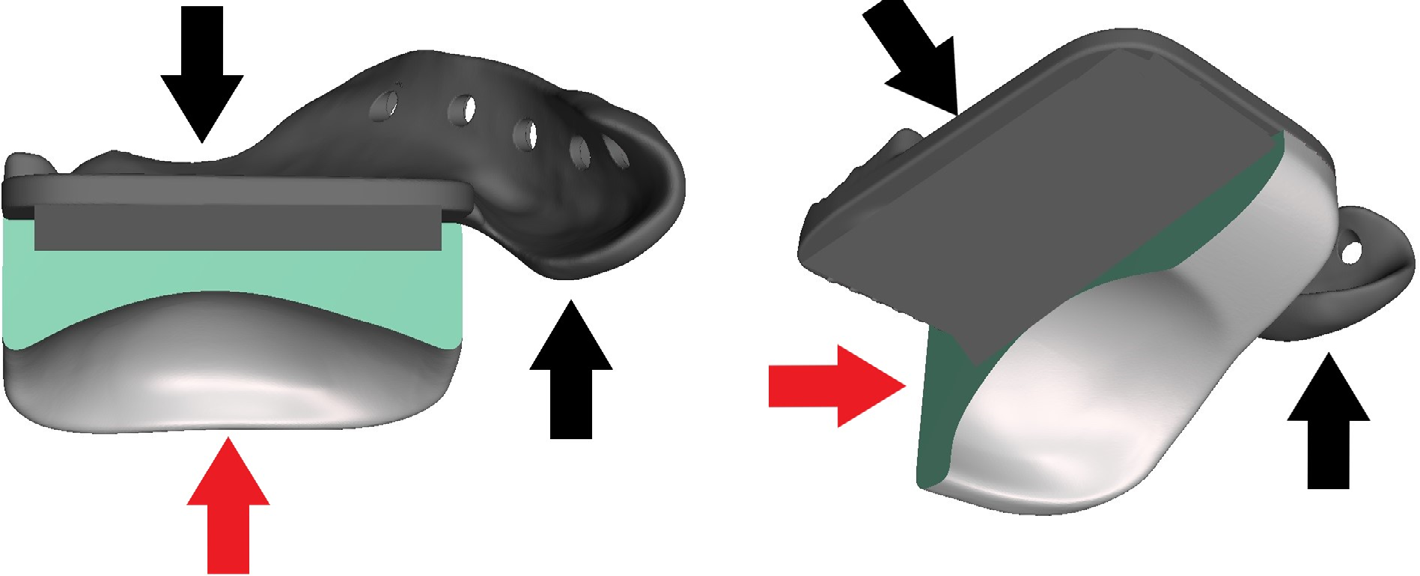
Fossa component with sagittal and transversal sectional view. The titanium mesh connecting the UHWMPE to the titanium component has been removed for proprietary reasons. (A) Frontal view. Black arrow: titanium component. Red arrow: UHMWPE component. (B) Inferior view. Black arrow: titanium component. Red arrow: UHMWPE component

The fossa component (Figure 2) consisted of an AM Ti_6_Al_4_V part (procedure as described above), which fits on the glenoid fossa and articular eminence, as well as a computer numerical controlled (CNC) milled vitamin E‐enriched UHMWPE part facing the artificial condyle. Details concerning the grade and manufacturing of the UHMWPE are proprietary information. Both parts were joined together by hot pressing a Ti_6_Al_4_V scaffold structure onto the UHMWPE. The fossa component was ɣ‐irradiated (100 kGy, Gammatom s.r.l.) to increase the number of crosslinks in the UHMWPE and for sterilization purposes. Ti alloy screws (Ti_6_Al_4_V grade 5, 2.0‐mm diameter; Surgi‐Tec NV) were used for fixation of the fossa component. Both the screws and condylar component were 40 min autoclaved in a 134°C–5 minutes cycle.

### 2.3 Surgical protocol

The left side of the face was aseptically prepared after orotracheal intubation and antibiotics (Enrofloxacin 5 mg/kg [Floxadil, EMDOKA BVBA]) were administered at the start of surgery, up until 5 days post‐operatively. The joint was accessed through both an incision over the posterior lower border of the mandible and a pre‐auricular S‐shaped incision inferior to the zygomatic arch. Once proper access was obtained, a patient/prosthesis specific Ti alloy (Ti_6_Al_4_V ELI, grade 23, CADskills BV) cutting guide was fixed onto the vertical ramus and a condylectomy with preservation of the LPM insertion was performed. This LPM insertion was isolated and a PDS 0 suture (Ethicon, Somerville) was threaded through the tendon of the LPM. After fitting a dummy version of the fossa component and adapting the soft tissues if needed, the fossa component was fixed to the zygomatic arch with five screws between 5 and 13 mm in length. Next, the PDS suture was run through the condylar tunnel and the ramal component was positioned and fixated onto the mandibular stump using seven screws between 13 and 17 mm in length. Important to remark were the difficulties faced to properly attach the LPM onto the scaffold, due to an obstructive edge at the anteromedial side of the UHMWPE part of the fossa component. Consequently, all UHMWPE parts were scalpel‐reduced at their non‐articulating anteromedial side. A multi‐layer closure was then performed, after which a compressive bandage was applied. A more detailed description of the surgery protocol can be found in one of our earlier published papers.^24^

### 2.4 Euthanasia and implant retrieval

Ten months after surgery, after being kept in an indoor pen, all 14 sheep were euthanized. All sheep were then decapitated and the left half of the skull was retained. The rest of the sheep was disposed of. Next, the left side of the skull was skinned, and the neurocranium, left eye, and anterior maxillary and mandibular halves were removed. After 3 months of immersion in formalin 4%, the peri‐articular tissues were resected for histological evaluation. The condyle was transected at the condylar neck by means of an Exakt 300 diamond band saw (EXAKT Technologies, Inc.) at Morphisto Gmbh. The fossa component was first clinically evaluated for its bony integration (e.g., if any macro‐motions were seen or if a fibrous layer had formed between the implant and the bone) after which the screws were removed and the fossa was removed from the skull.

With respect to the fossa, both linear and volumetric wear analysis of the articulating UHMWPE surface was performed by means of optical scanning. Linear wear, expressed in mm/year, is used in orthopedic surgery to determine the lifecycle of an implant. However, as it does not determine the total amount of UHMWPE volume that is lost, the volumetric wear, reported as mm^3^ per year, was evaluated as well. This is of importance, as it evaluates the total amount of debris that is formed and does not just evaluate the deepest point of material loss on the bearing surface.

To determine the amount of linear wear, first a 3D scanner applying blue‐light technology (ATOS CORE 135, GOM GmbH,) was used. The scanner was first calibrated according to the company prescribed calibration procedure, using a type CP40/170 calibration plate. This glass plate has circular markers with several markers having a larger diameter compared to the rest of the markers. These larger markers define the coordinate origin of the panel coordinate system. The 3D coordinates of the central points of each circular marker are measured, as well as distances between certain defined markers. This calibration process was performed and certified by a GOM‐employed specialist, resulting in a 13‐μm accuracy. However, because this 3D scanner does not allow for evaluation beyond a depth of 1 mm, the linear wear of these samples was recalculated and confirmed using a LC60Dx laser line scanner (LLS) (Nikon Metrology NV) mounted onto an MC16 Coordinate Measuring Machine (CMM) (Coord3 s.r.l.) through an indexable PH10M rotary head (Renishaw Benelux B.V.). This LLS system was also used to assess the volumetric wear of the UHMWPE fossa part. To this end, Focus Inspection version 9.4 (Nikon Metrology NV) was used to create an STL file of the point cloud generated through scanning the fossa with the LLS. Prior to scanning the fossa, all of the 21 kinematic error sources (the axes' translational, rotational and squareness error components) of the MC16 CMM were calibrated, to identify and compensate for any geometrical errors. This calibration was performed by a manufacturer technician, following a standardized method, reaching a micron level of precision for each individual axis. Furthermore, as to eliminate any environmental changes, all measurements were performed in a climate and humidity controlled room with air pressure monitoring. Lastly, prior to performing the CMM, qualification of the combined system of CMM and LLS was performed. This was done by use of a reference sphere, which was measured from all orientations used within the scanning sequence. The margin of error of this entire measurement technique is estimated to range from 0.01 to 0.1 mm. This generated STL was then overlapped with the STL of the design of the fossa component by means of a “best fit” iterative closest‐point algorithm using GOM Inspect (GOM GmbH). This method does not allow for closed loop information, as would be the case when reference points were marked before implantation ensuring a 100% fit. Instead, up to hundreds of matching points are calculated by the program's algorithm, in order to provide a reliable and reproducible overlap. For cooperative surfaces, this technique results in the same accuracy and error margin as provided by the scanner.

The “explanted‐STL” was then subtracted from the “design‐STL” to quantify the volume lost due to wear. Next, the articulating areas of the UHWMPE were isolated and evaluated rather than the entire UHMWPE fossa part. This was done to prevent overestimation of the wear volume, due to the scalpel reduction that was performed during implantation.

**FIGURE 3:**
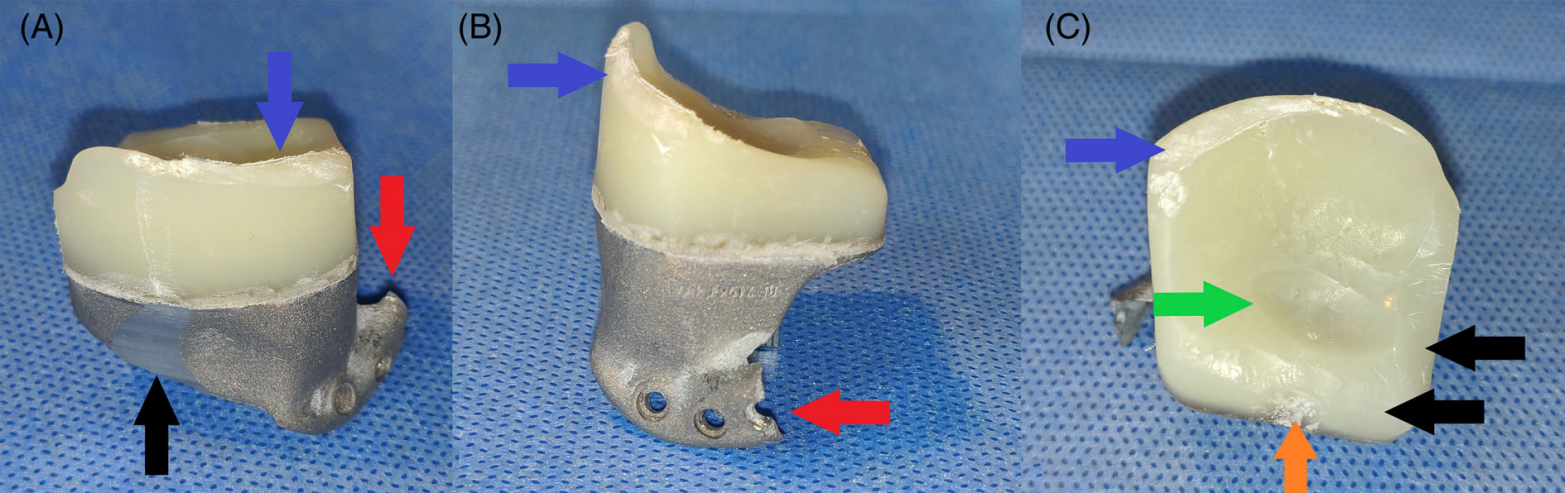
Macroscopic images of explanted fossa components of sheep #4473, with additional damage, having occurred during the explantation. This severe additional damage, occurred during retrieval, no longer allowed for reliable overlapping with the ‘design’‐STL. No linear, or volumetric wear could be analyzed of this fossa. (A) Posterior view. Blue arrow: damage to posterior UHMWPE ridge. Black arrow: damage to titanium part. Red arrow: damage to titanium extension for fixation onto the zygomatic arch. (B) Lateral view. Blue arrow: damage to posterior UHMWPE ridge. Red arrow: damage to titanium extension for fixation onto the zygomatic arch. (C) Inferior view. Blue arrow: damage to posterior UHMWPE ridge. Black arrow: scalpel‐reduced non‐articulating UHMWPE. Green arrow: worn out UHMWPE due to articulating with the condylar surface. Orange arrow: anteriorly worn out UHMWPE volume due to contact with the coronoid process

Wear volume was calculated using VGSTUDIO MAX Version 3.3.2 (Volume Graphics GmbH). The linear and volumetric UHMWPE one fossa could not be analyzed because the software was not able to retrieve a “best fit” between the design and the scan of the explanted fossa. The error margin in the overlap between the two STL models was too large to provide reliable results due to the intraoperative trimming of UHMWPE in non‐load‐bearing regions, as well as the posterior UHMWPE ridge being erroneously trimmed down during the post‐euthanasia implant retrieval as well as the titanium part for fixation onto the zygoma. (Figure 3) While this does not affect the articulating surface, the difference between the “design‐STL” and “explanted‐STL” was too significant for the best‐fit algorithm, thus resulting in non‐cooperative surfaces. One additional fossa could not be analyzed for volumetric wear because the software was unable to provide a “best fit” between both explanted fossa and their ‘design‐STL’, within the margin of error. As a result, a reliable volumetric wear volume could not be determined.

Both the non‐coated and coated Ti_6_Al_4_V condylar surfaces were evaluated using a 3D scanner (ATOS CORE 135, GOM GmbH) to determine the linear wear of the condylar articulating surface, in similar fashion to the UHMWPE fossa part.

The surface roughness was determined by 3D non‐contact profilometry using a confocal laser microscope (μSurf Explorer, NanoFocus AG). For each sample, a 4.5 × 1.5 mm^2^ worn area of the condylar surface was selected and polynomial filters were applied to remove form of the condyles. 3D surface roughness amplitude parameters (average roughness Sa, arithmetic mean of the absolute values of the surface departures from the mean plane, and root mean square height Sq, the root mean square value of the surface departures) were determined. In addition, a 2D profile was generated along the long direction of the scanned area (multiple profiles were extracted and averaged) and 2D surface roughness amplitude parameters were defined (average roughness Ra, the arithmetic average of the absolute values of the profile heights, and maximum height of the profile, Rt, the vertical distance between the highest and lowest points of the profile). One pristine (i.e., not implanted) coated condyle was assessed. It served as a reference for both the non‐coated and coated condyles as the application of the DLC coating does not alter the surface smoothness. In addition, the surface of both types of condyles was also investigated using a light microscope (Vertex 251UC, Micro‐Vu) at magnifications of 19×, 37×, 204×, and 425×. Furthermore, the surfaces of the DLC‐coated condyles were visualized using scanning electron microscopy (SEM, Nova NanoSEM 450, FEI Company) operated at standard high‐vacuum settings at 5 mm working distance and 10 keV accelerating voltage.

## 3 RESULTS

### 3.1 Analysis of the UHMWPE fossa component

**FIGURE 4:**
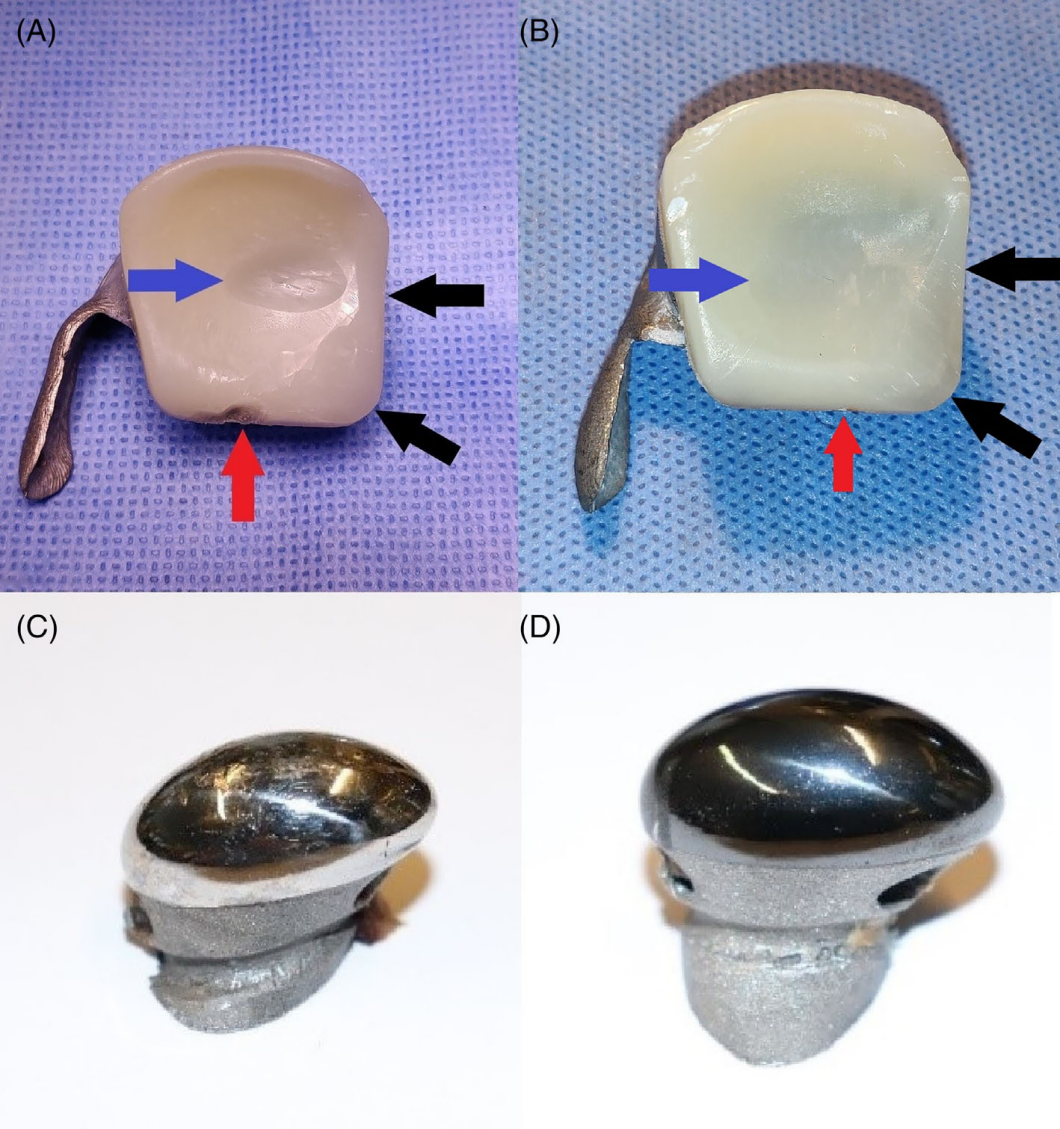
Representative macroscopic images of explanted components of the custom temporomandibular joint total joint replacement after 9 months of mastication and rumination in a sheep model. (A) Ultra‐high molecular weight polyethylene (UHMWPE) fossa of sheep #1724 that articulated with a non‐coated condyle. Blue arrow: worn out UHMWPE due to articulating with the condylar surface. Black arrow: scalpel‐reduced non‐articulating UHMWPE. Red arrow: anteriorly worn out UHMWPE volume due to contact with the coronoid process. (B) UHMWPE fossa of sheep #5158 that articulated with an HadSat® (H‐DLC) diamond‐like carbon coated condyle. Blue arrow: worn out UHMWPE due to articulating with the condylar surface. Black arrow: scalpel‐reduced non‐articulating UHMWPE. Red arrow: anteriorly worn out UHMWPE volume due to contact with the coronoid process. (C) Non‐coated Ti_6_Al_4_V condyle. (D) H‐DLC coated condyle

Macroscopically, all fossae exhibited UHMWPE wear in the center as well as in the middle of the anterior border, where the polyethylene came into contact with the coronoid process (Figure 4A,B). No macroscopically visible signs of UHMWPE delamination, warping, or fracturing were seen. There was some soft tissue adhesion on the medial and lateral side of the fossa, where the UHMWPE was pressed against the titanium, however upon closer inspection, this soft tissue adhesion remained strictly superficial and no dehiscence between the two components was seen macroscopically, nor during probing and removal of the soft tissue.

**FIGURE 5:**
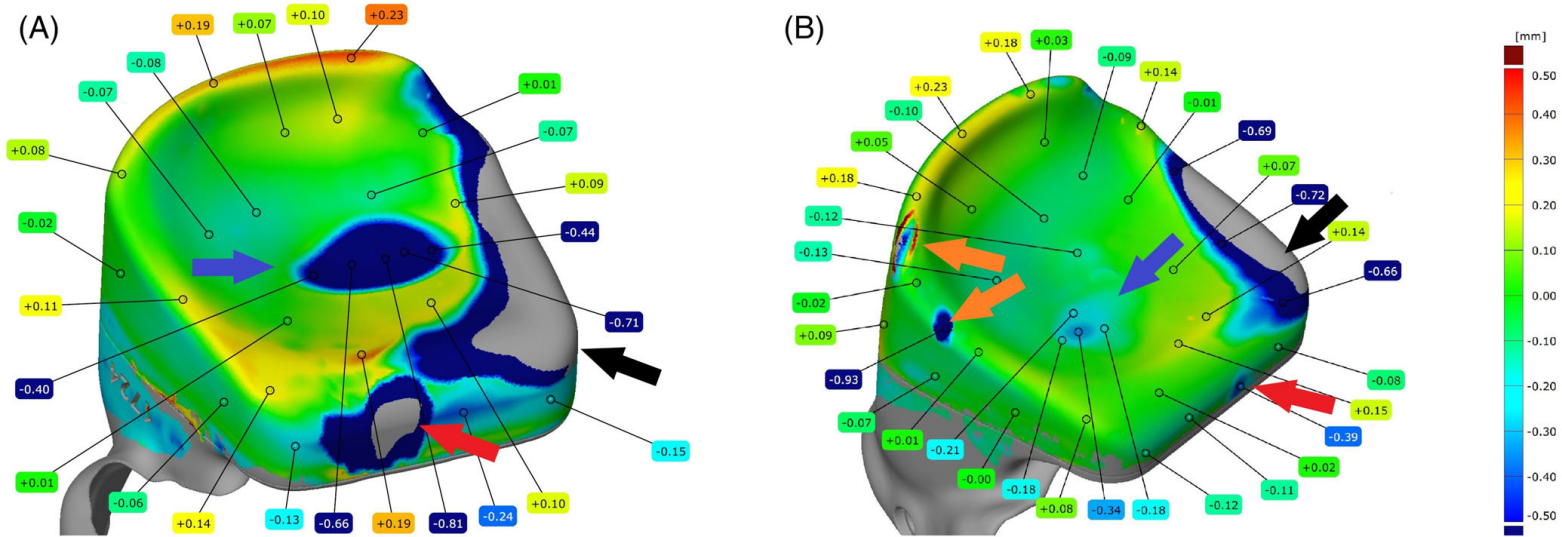
Representative 3D scans of explanted components of the custom temporomandibular joint total joint replacement after 9 months of mastication and rumination in a sheep model. (A) Ultra‐high molecular weight polyethylene (UHMWPE) fossa of sheep #1724 that articulated with a non‐coated condyle. The maximal wear depth measures 0.81 mm. Blue arrow: worn out UHMWPE due to articulating with the condylar surface. Black arrow: scalpel‐reduced non‐articulating UHMWPE. Red arrow: anteriorly worn out UHMWPE volume due to contact with the coronoid process. (B) UHMWPE fossa of sheep #5158 that articulated with an HadSat® diamond‐like carbon coated condyle. The maximal wear depth measures 0.34 mm. Blue arrow: worn out UHMWPE due to articulating with the condylar surface. Black arrow: scalpel‐reduced non‐articulating UHMWPE. Red arrow: anteriorly worn out UHMWPE volume due to contact with the coronoid process. Orange arrow: worn out sections due to post‐mortem dissection of the overlaying soft tissues

3D scanning of the fossa surface, articulating either with an uncoated (Figure 5A) or coated condyle (Figure 5B) was conducted and in most samples the wear volume clearly corresponded with the form of the condyle, with the articulation taking place in the center of the fossa. However, in sheep # 5158 the center of the wear volume was located slightly more laterally, whereas the mediolateral direction was slightly more diagonal compared to the other samples (Figure 5B).

**FIGURE 6:**
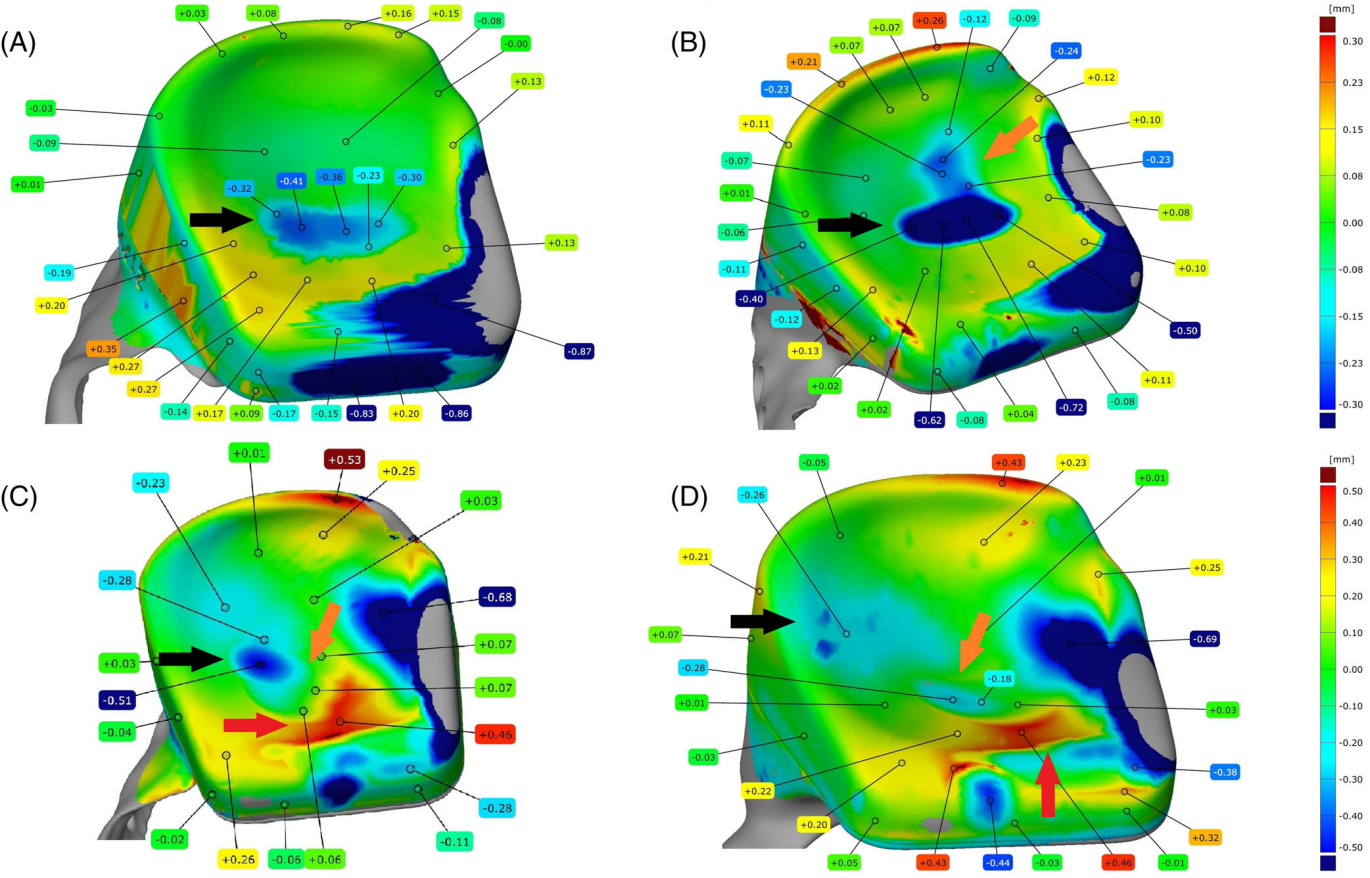
3D scans of explanted fossa component of the custom temporomandibular joint total joint replacement after 9 months of mastication and rumination in a sheep model with deviant wear patterns. (A) Ultra‐high molecular weight polyethylene (UHMWPE) fossa of sheep #2177 that articulated with a non‐coated condyle. Black arrow: worn out UHMWPE due to articulating with the condylar surface, with uneven edges. (B) UHMWPE fossa of sheep #4246 that articulated with an H‐DLC coated condyle. Black arrow: main worn out UHMWPE volume due to articulating with the condylar surface. Orange arrow: posteriorly orientated UHMWPE wear track. (C) UHMWPE fossa of sheep #8087 that articulated with an H‐DLC coated condyle. Black arrow: main worn out UHMWPE volume due to articulating with the condylar surface. Orange arrow: three additional condylar‐shaped UHMWPE wear tracks. Red arrow: UHMWPE creep deformation, in non‐articulating region. (D) UHMWPE fossa of sheep #7998 that articulated with a non‐coated condyle. Black arrow: main worn out UHMWPE volume due to articulating with the condylar surface. Orange arrow: initial, centered, UHMWPE wear volume due to articulating with the condylar surface. Red arrow: UHMWPE creep deformation, in non‐articulating region

In four sheeps, more apparent deviant wear patterns were found. (Figure 6A–D) The edges of the worn volume of ewe # 2177 were far less clearly marked compared to the other samples (Figure 6A). The fossa in sheep #4246 not only showed this distinct wear volume in the center, but also a slight additional posteriorly orientated wear track (Figure 6B). The fossa of ewe # 8087 showed one main wear volume, which was also more diagonally orientated and additionally three more anteriorly positioned wear “bodies” (Figure 6C). While no clear macroscopic signs of creep were seen, 3D surface analysis revealed some warping anteriorly of these additional wear bodies. Lastly, ewe # 7998 not only developed only little wear near the center of the fossa, but there also occurred wear near the posterior lateral border of the implant, as well as some warping, anteriorly from the center wear volume. (Figure 6D). Thus, in both cases showing warping, this occurred in non‐articulating locations.

While 3D scanning of the fossa surface seemed to indicate more extensive wear for UHMWPE components in contact with a non‐coated Ti_6_Al_4_V condyle as compared to a coated condyle (Figure 5A,B), no significant difference in the amount of linear, nor volumetric wear, was seen between both groups of fossa.

As already mentioned previously, due to not being able to determine the linear wear by means of 3D scanning for all the fossa, the amount of linear wear was determined by means of LLS. However, in one sample (ewe # 4473, Figure 4) no proper alignment of the explanted model and the STL file was possible and thus no (correct) measurement was possible. The average linear wear of the UHMWPE surface in contact with the non‐coated condyle was 0.88 ± 0.41 mm, while for the UHMWPE surface in contact with the coated condyle it was 0.67 ± 0.28 mm. The difference between these two groups was not statistically significant (*p* = .3765, *t*‐test). When converted to human mastication habits, these values are equivalent to 0.04 ± 0.02 mm respectively 0.03 ± 0.01 mm/year (Tables 1, 2, 3).

**TABLE 1:** Quantitative results of the damage analysis on explanted components of the custom TMJ TJR. For the UHMWPE fossa component, linear and volumetric wear were determined by 3D scanning and laser line scanning. For the Ti_6_Al_4_V condylar surface, surface roughness was assessed using 3D non‐contact profilometry. Prostheses incorporating a non‐coated Ti_6_Al_4_V condyle or a H‐DLC‐coated Ti_6_Al_4_V condyle are compared. Values represent mean ± standard deviation

	Non‐coated Ti_6_Al_4_V condyle	H‐DLC‐coated Ti_6_Al_4_V condyle
Linear wear of UHMWPE fossa		
Max wear (mm)	0.88 ± 0.41	0.67 ± 0.28
Maximal wear/year in sheep (mm/year)	1.11 ± 0.53	0.85 ± 0.35
Maximal wear/year in humans (mm/year)	0.04 ± 0.02	0.03 ± 0.01
Volumetric wear of UHMWPE fossa		
Total wear (mm^3^)	45.85 ± 22.01	25.29 ± 11.43
Wear/year in sheep (mm^3^/year)	58.17 ± 27.95	32.04 ± 14.49
Wear/year in humans (mm^3^/year)	2.08 ± 1.00	1.15 ± 0.52
Roughness of Ti_6_Al_4_V condyle		
Sa (μm)	2.40 ± 2.08^a^	0.69 ± 0.07^a^
Sq (μm)	3.47 ± 3.01^a^	0.90 ± 0.08^a^
Ra (μm)	0.28 ± 0.17^a^	0.12 ± 0.04^a^
Rt (μm)	1.91 ± 1.23^a^	0.65 ± 0.27^a^

*Notes*: Sa = average roughness, the arithmetic mean of the absolute values of the surface departures from the mean plane within the sampling area. Sq = root mean square height, the root mean square value of the surface departures within the sampling area. Ra = average roughness, the arithmetic average of the absolute values of the heights of the assessed profiles. Rt = maximum height of the profile, the vertical distance between the highest and lowest points of the assessed profiles.

a Statistically significant difference between coated and non‐coated condyles.

**TABLE 2:** Amount of linear ultra‐high molecular weight polyethylene wear

	Sample	Max wear (mm)	Maximal wear/year (mm/year) (sheep)	Maximal wear/year (mm/year) (human)
H‐DLC‐coated TMJR	3520	0.81	1.03	0.037
	8087	0.51	0.65	0.023
	2177	0.41	0.52	0.019
	5158	0.34	0.43	0.014
	2549	0.81	1.03	0.015
	4249	1.15	1.46	0.052
Non‐coated TMJR	0032	0.64	0.81	0.029
	7998	0.28	0.35	0.013
	4246	0.72	0.91	0.033
	1724	0.81	1.03	0.036
	8787	1.35	1.71	0.061
	4248	1.48	1.88	0.067
	4473	>1	> 1.27	> 0.045

*Notes*: For sample 4473, the error margin in the overlap between the two STL models was too large for the “best fit” iterative closest‐point algorithm to provide reliable results. Based on the 3D scanner analysis, the linear wear was found exceed 1 mm, yet no specific result was determined.

Abbreviation: TMJR: temporomandibular joint replacement.

**TABLE 3:** Amount of volumetric ultra‐high molecular weight polyethylene wear

	Sample	Total volumetric wear (mm^3^)	Volumetric wear/year (mm^3^/year) (Sheep)	Volumetric wear/year (mm^3^/year) (Human)
H‐DLC‐coated TMJR	4249	42.70	54.12	1.94
	2177	16.45	20.85	0.75
	3520	31.79	40.29	1.45
	2549	32.92	41.7	1.5
	5185	9.18	11.63	0.42
	8087	18.68	23.67	0.85
Non‐coated TMJR	1724	32.61	41.33	1.48
	4246	27.77	35.19	1.26
	8787	59.45	75.34	2.7
	0032	26.47	33.84	1.20
	4248	82.96	105.15	3.77
	7998	–	–	–
	4473	–	–	–

*Note*: For both sample 7998 and 4473, the error margin in the overlap between the two STL models was too large for the ‘best fit’ iterative closest‐point algorithm to provide reliable results.

Abbreviation: TMJR: temporomandibular joint replacement.

An average volume loss of 45.85 ± 22.01 mm^3^ could be observed for the UHMWPE articulated with the non‐coated Ti_6_Al_4_V condyle surface as compared to 25.29 ± 11.43 mm^3^ when articulated with the coated condyles. The difference was not statistically significant (*p* = .1448; *t*‐test). Based on these results, the amount of volumetric wear translates to 2.08 ± 1.00 resp. 1.15 ± 0.52 mm^3^/year of human mastication.

### 3.2 Analysis of the Ti_6_Al_4_V condylar component

**FIGURE 7:**
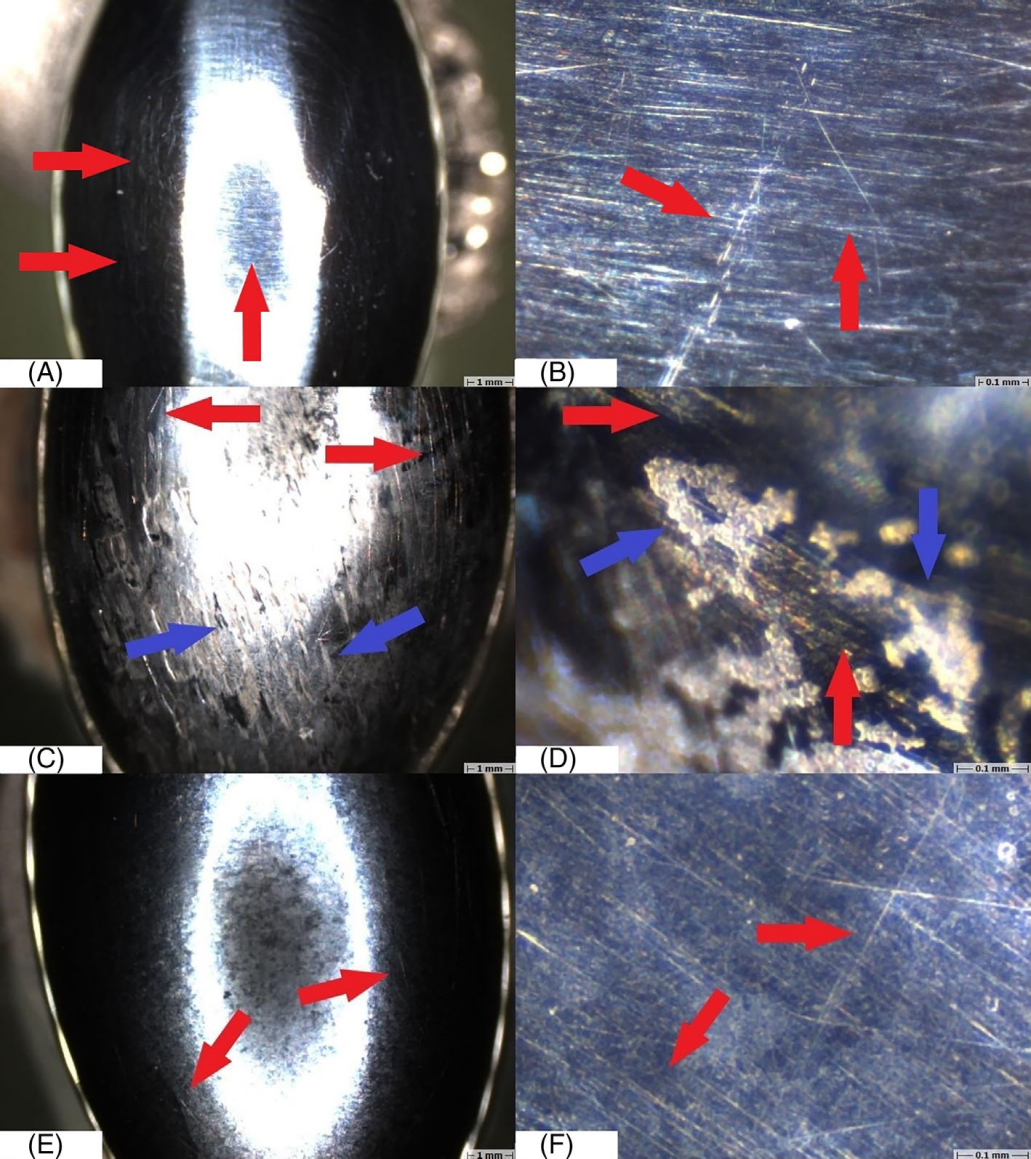
Representative light microscopy images of the condylar surface of the custom temporomandibular joint total joint replacement. (A,B) Condylar surface of a pristine, non‐coated condyle. Red arrow: superficial, multi‐directional scratches. (C,D) Condylar surface of the non‐coated condyle of sheep #8787, explanted after 9 months of mastication and rumination in a sheep model. Red arrow: superficial, multi‐directional scratches. Blue arrow: deeper abrasive wear. (E,F) Condylar surface of the HadSat® diamond‐like carbon‐coated Ti_6_Al_4_V condyle of sheep #5158, explanted after 9 months of mastication and rumination in a sheep model. Red arrow: superficial, multi‐directional scratches

**FIGURE 8:**
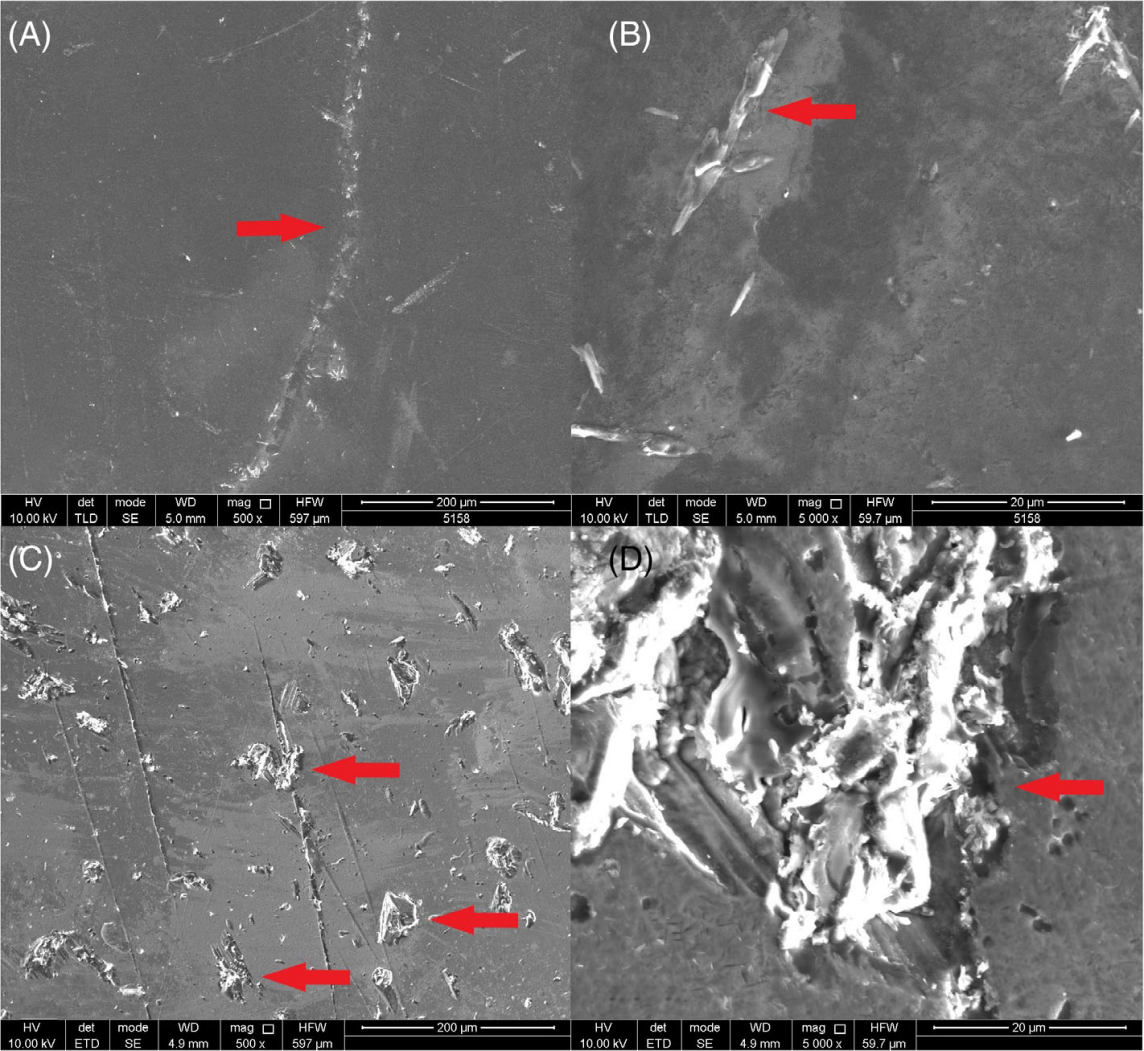
Scanning electron microscopy images of the coated condylar surfaces after explantation. (A) Sheep #5158 Intact, smooth, condylar surface without significant damage (magnification 500×). Red arrow: superficial scratch with intact coating. (B) Sheep #5158 intact, smooth, condylar surface without significant damage (magnification 5000×). Red arrow: superficial scratch with intact coating. (C) Sheep #2177 damaged condylar surface (magnification 500×). Red arrow: deep abrasive wear, penetrating the condylar coating. (D) sheep #2177 damaged condylar surface (magnification 5000×). Red arrow: deep abrasive scratches penetrating the condylar coating

Macroscopically, the non‐coated condyles exhibited a significant amount of surface damage, ranging from superficial scratches to deep pits, whereas on the coated condyles no obvious damage could be observed (Figure 4C,D). This was again confirmed by 3D scans of the condylar surfaces where pits and scratches could be observed in the center of the non‐coated condyles while the surface of the coated condyles appeared smooth. Microscopic investigation of the surface revealed multi‐directional surface scratches on both types of condyles, yet the scratches appeared remarkably deeper and more densely concentrated on the non‐coated Ti_6_Al_4_V condylar surfaces then on the H‐DLC‐coated surfaces (Figure 7B,C). For both types, the surface damage was limited to the load‐bearing surface of the condyle. In comparison to the pristine condyle, similar multi‐directional scratches were seen on the retrieved coated condyles, indicating that these scratches are due to the polishing protocol that is applied before coating the condyle (Figure 7A). The amount of surface marks found on the explanted non‐coated condyles was markedly higher, indicating that some abrasion had occurred during usage. For a more detailed investigation of the coated surfaces, SEM analysis was performed. This analysis confirmed that in five out of six condyles, multi‐directional scratches were present without significant damage to the articular surface (Figure 8A,B). The condylar surface of ewe #2177 presented deeper marks, for which an additional surface topography analysis using MeX (Alicona Imaging GmbH) was performed, revealing that the surface damage penetrated through the DLC coating (Figure 8C,D).

The surface roughness of the condylar bearing surface was analyzed using a confocal laser microscope. The 3D as well as 2D surface roughness amplitude parameters are presented in Tables 1 and 4. Overall, these quantitative results indicate that the roughness for the non‐coated Ti_6_Al_4_V condylar surface was higher than for the DLC‐coated Ti_6_Al_4_V condylar surface and analysis showed a statistically significant difference between both the coated and non‐coated average surface roughness for both *Sa* (p = 0.0083; Mann–Whitney U test) and *Ra* (p = 0.0182; Mann–Whitney U test).

**TABLE 4:** Condylar surface roughness analysis

Sample	Sa (μm)	Sq (μm)	Ra (μm)	Rt (μm)
2177	0.77	0.98	0.16	0.78
2549	0.61	0.81	0.10	0.81
3520	0.64	0.83	0.10	0.59
4249	0.70	0.91	0.10	0.69
5158	0.64	0.83	0.07	0.09
8087	0.78	1.02	0.18	0.91
Non‐implanted diamond‐like carbon	0.58	0.76	0.09	0.53
1724	2.30	3.38	0.20	1.52
4246	1.27	1.81	0.20	1.22
8787	6.91	10.1	0.63	4.65
0032	0.72	0.91	0.14	0.66
4248	1.05	1.80	0.26	2.01
7998	0.86	1.27	0.12	1.03
4473	3.72	5.05	0.44	2.31

*Notes*: Sa = average roughness, the arithmetic mean of the absolute values of the surface departures from the mean plane within the sampling area. Sq = root mean square height, the root mean square value of the surface departures within the sampling area. Ra = average roughness, the arithmetic average of the absolute values of the heights of the assessed profiles. Rt = maximum height of the profile, the vertical distance between the highest and lowest points of the assessed profiles.

**FIGURE 9:**
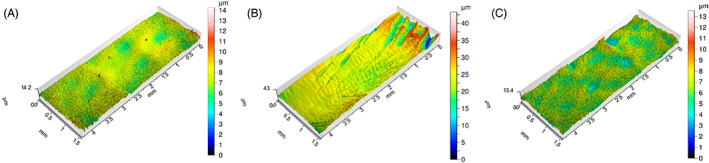
Representative 3D roughness profiles of the condylar surface of the custom temporomandibular joint total joint replacement. (A) Condylar surface of pristine, non‐implanted, coated condyle. (B) Condylar surface of a non‐coated condyle of sheep #1724, explanted after 9 months of mastication and rumination in a sheep model. (C) Condylar surface of a HadSat® diamond‐like carbon coated condyle of sheep #5185, explanted after 9 months of mastication and rumination in a sheep model

Moreover, comparison with the pristine DLC‐coated condyle demonstrates a similar surface roughness for DLC‐coated surface before and after 22 months of implantation in the sheep model. These results are also supported by a qualitative assessment of the three types of condylar surfaces, with their representative 3D roughness profiles shown in Figure 9.

## 4 DISCUSSION

The present study evaluated a novel model of TMJ TJR in a sheep model and set out to identify the wear patterns of both the condylar and fossa components of the prosthetic device implanted over a period of 288 days. This theoretically equals an estimated lifespan of 22 years in human implantation, based on the number of mastication movements.

While being an in vivo experiment, we were not constricted to the use of in vivo wear evaluation techniques such as the radiostereometric analysis introduced by Selvik et al.^26^ as the sheep were sacrificed and the TMJR were explanted. Thus, optical scanning was used to determine linear UHMWPE wear, while CMM laser scanning was used to determine volumetric UHMWPE wear and reconfirm the results on linear wear. The articulating Ti condylar surface was analyzed as well, by means of scanning electron and confocal laser microscopy surface.

### 4.1 UHMWPE wear analysis

Linear wear, expressed in mm/year, is used in orthopedic surgery to determine the lifecycle of an implant. It does not however determine the total amount of UHWMPE volume that is lost. This is of importance as, along with particle size and shape, the wear volume is a significant determinant for the occurrence of periprosthetic osteolysis.^8^ Dumbleton et al.^27^ concluded that the risk of osteolysis occurring is rare as long as the total amount of linear wear remains under 0.1 mm/year. Similar findings were reported by Oparaugo et al.,^28^ who found that the risk of osteolysis was rare if the maximal amount of wear was limited to 80 mm^3^/year.

Both the coated and non‐coated TMJR systems exhibited linear wear equivalent to less than 0.1 mm and volumetric wear equivalent of far less than 80 mm^3^/year of human functioning (Tables 1, 2, 3). In comparison to the average linear wear of 0.08–0.2 and 48–155 mm^3^/year volumetric wear per year in total hip implants and 0.05–0.23 mm linear wear per year for a total knee implant, our results can be considered excellent.^29^ Important to notice is that, while upon inspection, there was a qualitative difference observed between the fossa articulating with either a coated or non‐coated condyle, no statistically significant difference was observed between these samples. A Shapiro–Wilk test confirmed the Gaussian distribution of both the linear and volumetric wear data, supporting the use of a *t*‐test, yet post hoc power calculations indicated that this study would have needed 15 sheeps per group to achieve adequate power to detect a significant difference between these two groups of fossa. While the sample size of this study was chosen to minimize the number of animals subjected to, the invasive procedures required for this study it is highly likely that the non‐statistical difference that was found was due to the small group sizes.

Secondly, a displacement of the fossa was found in several ewes. While a 3‐month post‐operative CT scan revealed a good positioning of the fossa in ewe #7998, during the post‐mortem CT scan and dissection a significant caudodorsal displacement of the fossa was seen. This was also reflected by the wear pattern that was found through 3D scanning of the fossa component. In addition, sheep #5158 showed a normal positioning of the TMJR at 3 months after surgery, yet a limited latero‐inferior displacement of the fossa was found during the post‐operative dissection. A similar displacement was found in sheep #2177 at both the 6‐month post‐operative CT scan that was made as an exception, for a study analyzing the LPM insertion to the TMJR, as well as during explantation. However, as the displacement of the fossa was rather limited, this only led to a slightly more laterally positioned wear volume in case of ewe #5158 and the edges of the wear volume were less sharply marked in case of sheep #2177 (Figure 6). In addition to these three displaced fossa, also the fossa of ewe #4246 showed a deviant wear pattern, with a slight latero‐medial extension of the wear track. This could potentially be caused due to laterotrusive movements of the contralateral joint, with the implanted side functioning as stabilizing joint.

The displacement of these three fossa was most likely due to the use of 2 mm diameter screws for the fixation of the fossa component, as is done in human TMJ TJR. Keeping the higher mastication rate and laterotrusive movement in mind, the force the fossa is subjected to is higher compared to that in humans. This might have led to excessive stress in the bone surrounding the screws, resulting in bone resorption and micromovements between the fossa and the underlying bone, causing aseptic loosening of the implant component.^30^, ^31^, ^32^ In order to ascertain the effect of the altered wear patterns and volumes, the results of either only sheep # 7998 or all three sheeps were removed from the results and a renewed statistical evaluation was made. However, the difference in linear and volumetric wear between both groups remained non‐significant, and in both cases, the human equivalent for the measured linear and volumetric wear remained well within the acceptable range. Despite the deviant wear pattern for the fourth fossa, we kept these results included, as there was no displacement that occurred.

### 4.2 Condylar wear analysis

In knee and hip arthroplasty, there is an industry standard for surface smoothness (American Society for Testing and Materials F 2083‐12, American Society for Testing and Materials F 2033‐12), which does not exist for TMJ replacements. This is of importance because earlier studies have proven that a high surface roughness (Ra 0.2–0.63 μm) will also increase the amount of wear that can occur in the opposing articular surface^33^, ^34^, ^35^ and can lead to the formation of larger wear particles, which can cause third body wear.^14^, ^36^

In this study, the industry standard for total knee prostheses was applied to the TMJ implant surfaces. These surfaces were polished to obtain a Ra below 0.1 μm, which was confirmed by the surface roughness parameters determined here for DLC‐coated condyle prior to implantation (Ra = 0.09). The non‐coated implants exhibited a significant increase in wear after implantation, resulting in an Ra (0.28 ± 0.17) well above the orthopedic industry standard. The Ra of the DLC‐coated condyles (0.12 ± 0.04) however, remained well within the industry standard (Tables 1 and 4). Furthermore, the difference in both *Sa* and *Ra* was found to be significant by means of Mann–Whitney *U* test, as a non‐Gaussian distribution was found for the non‐coated condyles.

This non‐Gaussian distribution was due to the high Ra and Sa (6.59) that were measured for the condylar surface of ewe #8787. Despite the fossa being in its proper position, as well as the mandibular component, and although upon explantation no macroscopically visible third bodies were found inside the joint, the wear pattern on the condyle indicates third body abrasive wear occurred with the surface damage being mediolateral oriented. This is conformational to the expected mastication pattern, as sheep mainly perform laterotrusive movements. Due to this increased surface roughness, a high amount of linear (1.35 mm) and volumetric wear (59.45 mm^3^) was found in the fossa as well, supporting our statement for the importance of a low Ra and Sa in order to limit surface wear.^14^, ^36^ In order to evaluate the effect of this finding, a new Shapiro–Wilk test without this sample was performed, finding a Gaussian distribution for the other samples. However, a significant difference in both the Sa (*p* = .0692) and Ra (*p* = .0565) was still found when using an unpaired two sample *t*‐test, indicating a significant increase in *Sa* and *Ra* in the uncoated condyles, compared to the coated condylar surface.

### 4.3 Ti surface modification

Our in vivo results were also in line with several in vitro experiments, evaluating the amount of wear between DLC‐coated Ti as compared to non‐coated Ti articulating with UHMWPE, finding a decreased amount of wear in the former group.^37^, ^38^, ^39^ While these findings highlight the importance of Ti surface modification in load‐bearing surfaces, potential disadvantages have to be evaluated as well. A significant potential disadvantage to the use of a DLC coating is the relatively poor adhesion between the DLC layer and the Ti surface.^37^, ^40^, ^41^, ^42^, ^43^ This can lead to plastic deformation of the softer Ti when the implant is subjected to high forces. This in turn can lead to chipping or delamination of the DLC coating,^38^, ^40^, ^43^ which may result in a significant increase in Ra and subsequent wear. Other surface modification techniques, such as titanium nitride (TiN) coatings, also have this limitation, as delamination and third body wear can occur after physical vapor deposition (PVD) of the TiN coating.^14^, ^44^, ^45^ Several techniques have been developed to overcome this problem. One technique involves the use of a gradient coating in which the carbon concentration increases towards the surface. Another technique is to use plasma nitriding on the Ti first, and then apply the DLC coating through magnetron sputtering.^37^, ^43^ In this study, this limitation was addressed by using the patented HadSat®‐coating; no delamination was observed on the surfaces of any of the coated condyles.

### 4.4 Limitations

In total hip prostheses, the unworn volume of the acetabular component can be reconstructed when conducting a CMM measurement, out of an unworn surface, no such application exists at this moment for reconstruction of the fossa.^7^ Thus it would have been preferable to scan the pre‐wear UHMWPE component of the fossa before implantation, to limit any error margin. However due to sterilization issues, it was not achievable to scan the fossa after production. However, this error margin did not significantly affect the UHWMPE fossa part under investigation, as they were oversized 3D‐printed and consequently milled down to the original STL file boundaries with a precision of 0.02 mm, as was also the case for the titanium condylar component. In addition, as we were not able to scan the implants prior to implantation, we were unable to predetermine reference points as to use a closed loop information system to overlap the “pre‐implantation” STL and “explanted‐STL” and instead relied on the “best‐fit” method using GOM Inspect (GOM GmbH).

A second limitation we faced, were the fitting difficulties of the UHMWPE fossa during implantation, resulting in the trimming down of the non‐load‐bearing UHMWPE surfaces. While this allowed for easier implantation, this did result in problems determining the both linear and volumetric wear in one sample and volumetric wear in one additional sample. This was due to the ‘best‐fit’ algorithm no longer being able to find a sufficient amount of matching surface points between the design‐STL and the explanted fossa.

A significant limitation we were confronted with as well, was the lack of prior research into both in vitro and in vivo wear analysis in TMJ TJR. Thus, we were forced to compare our results to wear evaluation in total knee replacement (TKR).

## 5 CONCLUSION

Our custom additively manufactured TMJ replacement system is well‐suited for implantation, with an average linear and volumetric UHMWPE wear well below the maximum allowed per year in TKR, for both the non‐coated and H‐DLC‐coated Ti_6_Al_4_V condyles. Furthermore, the use of the H‐DLC coating significantly improved the surface roughness of the condylar surface. Based on these findings, the combined use of the condylar H‐DLC‐coating with Vitamin E‐stabilized UHMWPE should be considered the preferable TMJ implant option.

## CONFLICT OF INTEREST

Maurice Y. Mommaerts is co‐owner and innovation manager at CADSkills BV. Stijn Huys is R&D Officer at CADSkills BV.

## Data Availability

The data that support the findings of this study are available on request from the corresponding author. Some data concerning the production processes of the implants can not be shared, as well as some of the medication used during euthanasia, as this is proprietary information. This has also been mentioned in the manuscript. The data are not publicly available due to privacy or ethical restrictions.

## References

[1] Onoriobe U , Miloro M , Sukotjo C , Mercuri LG , Lotesto A , Eke R . How many temporomandibular joint total joint alloplastic implants will be placed in the United States in 2030? J Oral Maxillofac Surg. 2016;74:1531‐1538. doi:10.1016/j.joms.2016.04.011 27186874

[2] De Meurechy N , Braem A , Mommaerts MY . Biomaterials in temporomandibular joint replacement: current status and future perspectives—a narrative review. Int J Oral Maxillofac Surg. 2018;47:518‐533. doi:10.1016/j.ijom.2017.10.001 29126692

[3] Geetha M , Singh AK , Asokamani R , Gogia AK . Ti based biomaterials, the ultimate choice for orthopaedic implants—a review. Prog Mater Sci. 2009;54:397‐425. doi:10.1016/j.pmatsci.2008.06.004

[4] Evans JT , Walker RW , Evans JP , Blom AW , Sayers A , Whitehouse MR . How long does a knee replacement last? A systematic review and meta‐analysis of case series and national registry reports with more than 15 years of follow‐up. Lancet. 2016;393:655‐663. doi:10.1016/S0140-6736(18)32531-5 PMC6381229 30782341

[5] van Loon JP , de Bont LGM , Stegenga B , Spijkervet FKL , Verkerke GJ . Groningen temporomandibular joint prosthesis. Development and first clinical application. Int J Oral Maxillofac Surg. 2002;31:44‐52. doi:10.1054/ijom.2001.0175 11936399

[6] Sinno H , Tahiri Y , Gilardino M , Bobyn D . Engineering alloplastic temporomandibular joint replacements. McGill J Med. 2010;13:63‐72. PMC3277342 22363183

[7] Sagbas B , Durakbasa MN . Measurement of wear in orthopedic prosthesis. Proc Int Cong Adv Appl Phys Mater Sci. 2012;121:131‐134.

[8] Jacobs JJ , Roebuck KA , Archibeck M , Hallab NJ , Glant TT . Osteolysis: basic science. Clin Orthop Relat Res. 2001;393:71‐77. doi:10.1097/00003086-200112000-00008 11764373

[9] Alonso A , Kaimal S , Look J , et al. A quantitative evaluation of inflammatory cells in human temporomandibular joint tissues from patients with and without implants. J Oral Maxillofac Surg. 2009;67:788‐796. doi:10.1016/j.joms.2008.09.010 19304036

[10] De Meurechy N , Mommaerts MY . Alloplastic temporomandibular joint replacement systems: a systematic review of their history. Int J Oral Maxillofac Surg. 2018;47:743‐754. doi:10.1016/j.ijom.2018.01.014 29433767

[11] Elledge R , Mercuri LG , Attard A , Green J , Speculand B . Review of emerging temporomandibular joint total joint. Br J Oral Maxillofac Surg. 2019;57:722‐728. doi:10.1016/j.bjoms.2019.08.009 31455594

[12] van Loon JP , de Bont LGM , Spijkervet FKL , Verkerke GJ , Liem RSB . A short‐term study in sheep with the Groningen temporomandibular joint prosthesis. Int J Oral Maxillofac Surg. 2000;29:315‐324. doi:10.1016/S0901-5027(00)80044-2 11071231

[13] Van Loon JP , Verkerke GJ , de Bont LGM , Liem RSB . Wear‐testing of a temporomandibular joint prosthesis: UHMWPE and PTFE against a metal ball, in water and in serum. Biomaterials. 1999;20:1471‐1478. doi:10.1016/S0142-9612(99)00042-3 10458560

[14] Kerwell S , Alfaro M , Pourzal R , et al. Examination of failed retrieved temporomandibular joint (TMJ) implants. Acta Biomater. 2016;32:324‐335. doi:10.1016/j.actbio.2016.01.001 26768232

[15] van Loon JP , de Bont LGM , Boering G . Evaluation of temporomandibular joint prostheses. Review of the literature from 1946 to 1994 and implications for future prosthesis designs. J Oral Maxillofac Surg. 1995;53:984‐996. doi:10.1016/0278-2391(95)90110-8 7643290

[16] Ingawalé SM , Goswami T . Biomechanics of the temporomandibular joint. In: Goswami T , ed. Human Musculoskeletal Biomechanics. Croatia: InTech. 2012;1:159‐182. doi:10.5772/337021

[17] Angelo DF , Morouço P , Alves N , et al. Choosing sheep (*Ovis aries*) as animal model for temporomandibular joint research: morphological, histological and biomechanical characterization of the joint disc. Morphologie. 2016;100:223‐233. doi:10.1016/j.morpho.2016.06.002 27450042

[18] Almarza AJ , Brown BN , Arzi B , et al. Preclinical animal models for temporomandibular joint tissue engineering. Tissue Eng Part B Rev. 2018;24:171‐178. doi:10.1089/ten.TEB.2017.0341 29121815 PMC5994143

[19] Domingue BM , Dellow DW , Barry TN . The efficiency of chewing during eating and ruminating in goats and sheep. Br J Nutr. 1991;65:355‐363. 1878355 10.1079/bjn19910096

[20] Mommaerts MY . On the reinsertion of the lateral pterygoid tendon in total temporomandibular joint replacement surgery. J Cranio‐Maxillo‐Facial Surg. 2019;47:1913‐1917. doi:10.1016/j.jcms.2019.11.018 31810846

[21] Singh R , Dahotre NB . Corrosion degradation and prevention by surface modification of biometallic materials. J Mater Sci Mater Med. 2007;18:725‐751. doi:10.1007/s10856-006-0016-y 17143737

[22] Bracco P , Oral E . Vitamin E‐stabilized UHMWPE for total joint implants: a review. Clin Orthop Relat Res. 2011;469:2286‐2293. doi:10.1007/s11999-010-1717-6 21132413 PMC3126938

[23] Wolf C , Lederer K , Muller U . Tests of biocompatibility of alpha‐tocopherol with respect to the use as a stabilizer in ultrahigh molecular weight polyethylene for articulating surfaces in joint endoprostheses. J Mater Sci Mater Med. 2002;13:701‐705. 15348580 10.1023/a:1015750112343

[24] De Meurechy N , Verwilghen D , De Brucker Y , Van Thielen B , Mommaerts MY . Lateral pterygoid muscle enthesis reconstruction in total temporomandibular joint replacement: an animal experiment with radiological correlation. J Cranio‐Maxillo‐Facial Surg off Publ Eur Assoc Cranio‐Maxillo‐Facial Surg. 2021;49:256‐268. doi:10.1016/j.jcms.2021.01.029 33622558

[25] Verplancke K , De Waele W , De Bruyn H . Dental implants, what should be known before starting an in vitro study. Conf Sustainable Constr Des. 2011;2:360‐369. doi:10.1016/j.ecolecon.2014.05.003

[26] Selvik G . Roentgen stereophotogrammetric analysis. Acta Radiol. 1990;31:113‐126. 2196921

[27] Dumbleton JH , Manley MT , Edidin AA . A literature review of the association between wear rate and osteolysis in total hip arthroplasty. J Arthroplast. 2002;17:649‐661. doi:10.1054/arth.2002.33664 12168184

[28] Oparaugo PC , Clarke IC , Malchau H , Herberts P . Correlation of wear debris‐induced osteolysis and revision with volumetric wear‐rates of polyethylene: a survey of 8 reports in the literature. Acta Orthop Scand. 2001;72:22‐28. doi:10.1080/000164701753606644 11327409

[29] Benjamin J , Szivek J , Dersam G , Persselin S , Johnson R . Linear and volumetric wear of tibial inserts in posterior cruciate‐retaining knee arthroplasties. Clin Orthop Relat Res. 2001;392:131‐138. 10.1097/00003086-200111000-00016 11716374

[30] Chaudhary N , Lovald ST , Wagner J , Khraishi T , Baack B . Experimental and numerical modeling of screws used for rigid internal fixation of mandibular fractures. Model Simul Eng. 2008;2008:1‐11. doi:10.1155/2008/628120

[31] Tada S , Stegaroiu R , Kitamura E , Miyakawa O , Kusakari H . Influence of implant design and bone quality on stress/strain distribution in bone around implants: a 3‐dimensional finite element analysis. Int J Oral Maxillofac Implants. 2003;18:357‐368. 12814310

[32] Skinner R , Maybee J , Transfeldt E , Venter R , Chalmers W . Experimental pullout testing and comparison of variables in transpedicular screw fixation. A biomechanical study. Spine. 1976;1990(15):195‐201. doi:10.1097/00007632-199003000-00007 2353256

[33] Wang A , Polineni VK , Stark C , Dumbleton JH . Effect of femoral head surface roughness on the wear of ultrahigh molecular weight polyethylene acetabular cups. J Arthroplast. 1998;13:615‐620. doi:10.1016/S0883-5403(98)80002-8 9741435

[34] Jedenmalm A , Affatato S , Taddei P , et al. Effect of head surface roughness and sterilization on wear of UHMWPE acetabular cups. J Biomed Mater Res A. 2009;90:1032‐1042. doi:10.1002/jbm.a.32161 18671264

[35] Tubb CC , Longaray JA , Herrera L , Essner AP , Molloy RM . The effect of femoral component roughness on the wear of total knee implants. Orthop Res Soc. 2011;36.

[36] McGloughlin TM , Kavanagh AG . Wear of ultra‐high molecular weight polyethylene (UHMWPE) in total knee prostheses: a review of key influences. Proc Inst Mech Eng Part H, J Eng Med. 2000;214:349‐359. doi:10.1243/0954411001535390 10997056

[37] Jiang SW , Jiang B , Li Y , Li YR , Yin GF , Zheng CQ . Friction and wear study of diamond‐like carbon gradient coatings on Ti_6_Al_4_V substrate prepared by plasma source ion implant‐ion beam enhanced deposition. Appl Surf Sci. 2004;236:285‐291. doi:10.1016/j.apsusc.2004.04.032

[38] Kim DH , Kim HE , Lee KR , Whang CN , Lee IS . Characterization of diamond‐like carbon films deposited on commercially pure Ti and Ti‐6Al‐4V. Mater Sci Eng C. 2002;22:9‐14. doi:10.1016/S0928-4931(02)00106-6

[39] Firkins P , Hailey JL , Fisher J , Lettington AH , Butter R . Wear of ultra‐high molecular weight polyethylene against damaged and undamaged stainless steel and diamond‐like carbon‐coated counterfaces. J Mater Sci Mater Med. 1998;9:597‐601. doi:10.1023/A:1008917727604 15348693

[40] Roy RK , Lee K‐R . Biomedical applications of diamond‐like carbon coatings: a review. J Biomed Mater Res B Appl Biomater. 2007;83:72‐84. doi:10.1002/jbm.b.30768 17285609

[41] Liu X , Chu PK , Ding C . Surface modification of titanium, titanium alloys, and related materials for biomedical applications. Mater Sci Eng R Rep. 2004;47:49‐121. doi:10.1016/j.mser.2004.11.001

[42] Ching HA , Choudhury D , Nine J , Abu Osman NA . Effects of surface coating on reducing friction and wear of orthopaedic implants. Sience Technol Adv Mater. 2014;15(1):1‐21. doi:10.1088/1468-6996/15/1/014402 PMC5090599 27877638

[43] Yetim AF , Celik A , Alsaran A . Improving tribological properties of Ti_6_Al_4_V alloy with duplex surface treatment. Surf Coat Technol. 2010;205:320‐324. doi:10.1016/j.surfcoat.2010.06.048

[44] Van Hove RP , Sierevelt IN , Van Royen BJ , Nolte PA . Titanium‐nitride coating of orthopaedic implants: a review of the literature. Biomed Res Int. 2015;2015:1‐9. doi:10.1155/2015/485975 PMC4637053 26583113

[45] Mishnaevsky L , Levashov E , Valiev RZ , et al. Nanostructured titanium‐based materials for medical implants: modeling and development. Mater Sci Eng R Rep. 2014;81:1‐19. doi:10.1016/j.mser.2014.04.002

